# Anti-Zic4- and Anti-NMDAR-Positive Patients and Their Correlation with Clinical Course and Image Diagnostic Findings—Case Reports

**DOI:** 10.3390/diagnostics16152304

**Published:** 2026-07-23

**Authors:** Milen Hristozov, Krasimir Shukerski, Antoniya Yaneva, Marianna Murdjeva

**Affiliations:** 1 Department of Medical Microbiology and Immunology “Prof. Dr. Elissay Yanev”, Medical University of Plovdiv, 4002 Plovdiv, Bulgaria; 2 Research Institute, Medical University of Plovdiv, 4002 Plovdiv, Bulgaria; 3 Department of Neurology, Medical University of Plovdiv, 4002 Plovdiv, Bulgaria; krasimir.shukerski@mu-plovdiv.bg; 4 Neurology Clinic, “St. George” University Hospital Plovdiv, 4001 Plovdiv, Bulgaria; 5 Department “Medical Informatics, Biostatistics and E-Learning”, Medical University of Plovdiv, 4002 Plovdiv, Bulgaria; antoniya.yaneva@mu-plovdiv.bg; 6 Department of Microbiology and Virology, Medical University of Pleven, 5800 Pleven, Bulgaria; mariana.murdjeva@mu-pleven.bg; 7 Institute of Innovation and Smart Technology, University of Telecommunication and Posts, 1700 Sofia, Bulgaria

**Keywords:** paraneoplastic neurological syndromes, malignant neoplasia, antineuronal antibodies, paraneoplastic cerebellar degeneration, autoimmune encephalitis, case series

## Abstract

Background/Objectives: Paraneoplastic Neurological Syndromes (PNSs) comprise a rare group of disorders present in various types of malignant neoplasia. In most cases, PNSs precede the clinical symptoms and diagnosis of a tumor by years. It is therefore vital to recognize these neurological disorders, as early diagnosis of the neoplasia offers the opportunity for timely initiation of treatment and may improve patient outcomes. Case Presentation: Panels of antineuronal antibodies (ANeAs) were used to examine serum and cerebrospinal fluid (CSF) samples from two PNS patients using indirect immunofluorescence assay (IIFA) and immunoblotting (IB). Both patients were anti-Zic4-positive. In addition, one patient was anti-Zic4- and anti-NMDAR-positive. The individuals also exhibited abnormalities on computed tomography (CT) and magnetic resonance imaging (MRI)—focal lesions suggestive of paraneoplastic cerebellar degeneration (PCD) and a tumor. During follow-up neuroimaging examinations, no tumor was found. Discussion: An in-depth study of a case series of PNS patients was carried out. Anti-Zic4 was correlated with PCD, whereas anti-NMDAR was correlated with autoimmune anti-NMDAR encephalitis. These case series may represent a possible non-paraneoplastic cerebellar syndrome associated with autoimmune encephalitis. Moreover, it is necessary for such individuals to undergo long-term follow-up in order to facilitate the early detection of a possible malignant neoplasia. Conclusions: In the current study, we focus on a case series of two patients with severely impaired neurological status, abnormalities in CT and MRI suggestive of PCD, and positive results for ANeAs—anti-Zic4 and anti-NMDAR. Through this study, we aim to emphasize the need for thorough clinical and laboratory follow-up of patients for eventual tumor development.

## 1. Introduction

### 1.1. General Characteristics of PNSs

PNSs are widely accepted as immune-mediated diseases of the nervous system that occur as an indirect effect of malignancy and are triggered when tumors express proteins antigenically identical to those of neurons. These neuronal antigens are important for neural development and for the function and maintenance of phenotypes [1,2,3,4,5,6]. High-titer ANeAs targeting intracellular neuronal antigens (so-called “onconeuronal antigens”) have been identified and are associated with an underlying malignancy in more than 95% of cases [7]. Due to their high specificity (>90%), well-characterized antineuronal autoantibodies detected in serum and/or CSF serve as useful diagnostic biomarkers for the paraneoplastic origin of neurological syndromes [4,5,8,9,10,11]. Early recognition of PNSs is of the utmost importance, and screening for occult malignancies should not be limited to patients with classical syndromes of the central nervous system (CNS) [12]. In some cases, ANeAs may indicate the presence of specific tumor types even in the absence of clinical symptoms [4,5,9], facilitating faster diagnosis and earlier initiation of immunomodulatory treatment, which is also associated with better patient prognosis and disease outcome [8,11,12].

In studies involving live cultured neurons, it has been demonstrated that ANeAs do not bind to the targeted intracellular antigens [13]. Biopsy and autopsy materials from patients demonstrate induction of inflammatory infiltrates of T-cytotoxic lymphocytes localized around neurons, generation of a T-cell-mediated immune response, and neuronal degeneration (perforin or granzyme B cytotoxic mechanism) [14,15,16]. These factors often lead to irreversible neuronal damage, severe patient disability, and death, which explains the poor therapeutic response to immunotherapy in patients with PNSs [4,5,7,17].

### 1.2. Characteristics of Anti-Zic4 Antibodies, Clinical Association, and Prognosis

PCDs comprise a heterogeneous group of disorders characterized by subacute cerebellar ataxia, specific tumor types, and association with various ANeAs. Most of these autoantibodies are “onconeuronal”, with antibodies targeted against cell surface neuronal antigens being reported less frequently. PCD can be associated with any tumor type; however, the most common types of malignant neoplasia are lung cancer (typically SCLC, 33%), ovarian cancer (25%), breast cancer, and lymphomas (particularly Hodgkin lymphoma, 15%) [4,6,18]. In 60–70% of patients, neurological symptoms precede oncological symptoms by several months to 2–3 years and lead to early diagnosis of the tumor [4].

Anti-Zic4 (Zinc finger protein 4) paraneoplastic ANeAs are significantly associated with SCLC, and the classical clinical correlation in these patients is paraneoplastic encephalomyelitis (PEM), presenting as cerebellar dysfunction [19,20]. In some cases, anti-SOX1 antineuronal antibodies can also be detected in SCLC patients with PEM [19].

In one study, anti-Zic4 antibodies were identified in 61 patients with paraneoplastic neurological disorders (PNDs) or cancer. These antibodies were not detected in any of the 175 patients from groups with neurological disorders unrelated to cancer and the control group of healthy individuals. The patients in most cases also possessed anti-Hu or anti-CV2/CRMP5 antibodies [20].

Anti-Zic4, anti-Ma2, and anti-VGCC are found in nearly 40% of patients with PCD and SCLC [4]. Anti-Zic4 ANeAs are more common in PCD and SCLC and less common in ovarian carcinoma [20,21,22]. The associated PCD with the presence of anti-Zic4 antibodies is rare and usually exhibits a benign clinical course.

Although anti-Zic4, anti-Hu, and anti-CRMP-5 can be detected in patients with and without PND, in the study by Bataller et al. [20], the simultaneous presence of the antineuronal antibodies anti-Hu and anti-Zic4 was significantly associated with PND. A total of 26 patients positive for both antibodies developed PND before tumor diagnosis, and 8 patients developed the condition after tumor diagnosis. In comparison, only 5 patients positive for anti-Zic4 developed PND before tumor diagnosis, and 8 patients positive for anti-Zic4 developed the condition after tumor diagnosis [20]. Loehrer et al. [18] presented the first clinical case of rapidly progressive PCD and rhombencephalitis with detected anti-Zic4 antibodies. Patient excitation was attributed to autonomic dysfunction, which highlights the significance of anti-Zic4-associated neurological disorders [18].

### 1.3. Characteristics of Anti-NMDAR Antibodies, Clinical Association, and Prognosis

Antibodies to synaptic receptors or trans-synaptic protein complexes, e.g., N-methyl-D-aspartate (NMDA) receptors, glutamate receptors, Gamma-aminobutyric acid type b (GABAb) receptors, or Leucine-rich, glioma inactivated 1 (LGI1) protein, may cause neuronal damage or dysfunction in patients with LE. Antibodies to voltage-gated potassium channels (VGKCs) or to glutamate receptors may directly damage Purkinje cells in some patients with PCD [2].

Patients with anti-NMDAR encephalitis are typically hospitalized for prolonged periods (weeks to months) in intensive care units, with the development of seizures, reduced verbal output, and highly characteristic abnormal movements (orofacial dyskinesias and limb dyskinesias) [23,24,25,26].

Adult male patients initially present with generalized seizures, followed by focal seizures and, after several days, psychiatric or cognitive symptoms. Brain MRI findings are typically normal, and tumors are rarely found in these individuals. Testing for anti-NMDAR antibodies should be considered to avoid delays in initiating treatment [27].

The characteristic abnormal movements can fluctuate over a period of time [24]. Although this fluctuation phenomenon is rarely emphasized in literature sources, it may represent a useful diagnostic feature [28].

Seizures are most often characterized as focal, with or without loss of consciousness [29]. Detection of anti-NMDAR or other cell surface antibodies is considered confirmatory for the diagnosis of autoimmune encephalitis. This finding is important because adult patients respond well to immunotherapy [30].

Seizures are triggered by a specific autoimmune mechanism that typically exhibits a favorable response to therapy [31]. Patients with anti-NMDAR encephalitis should undergo long-term follow-up (at least 1 year) before considering a diagnosis of epilepsy in those who have seizures or require antiepileptic drugs [31]. Long-term follow-up demonstrates that in most patients, seizures resolve after the resolution of encephalitis. de Bruijn et al. [32] examined a series of clinical cases with one or more of the following symptoms: tonic–clonic seizures, focal seizures without or with impaired consciousness, status epilepticus, and refractory status epilepticus [32]. No patients suffered seizures after a median follow-up of 31 months [32]. In another study by Liu et al. [33], no patients suffered seizures at the second year of follow-up [33]. At the last follow-up, most patients were asymptomatic and were not receiving antiepileptic therapy [32]. The absence of seizures was attributed to immunotherapy in 47% of patients and antiepileptic drugs in 16%; in the remaining patients, the reason for seizure resolution was unclear (combined effects of immunotherapy and antiepileptic drugs or spontaneous improvement) [32]. The duration of antiepileptic therapy did not change the seizure-free period [33]. These findings support the gradual cessation of antiepileptic therapy during the convalescent stage [32,33].

### 1.4. Clinical Significance of ANeAs in PNSs

The practical clinical significance of ANeAs is that they significantly increase the index of suspicion for a paraneoplastic disorder and the type of antibody can help guide targeted screening for the associated tumor. ANeA tests have practical limitations. Considerable heterogeneity exists, as a given clinical syndrome (e.g., cerebellar degeneration) may be associated with one of several autoantibodies. In addition, a given autoantibody (e.g., anti-Hu) may be associated with different clinical manifestations. For most neurological syndromes, some patients possess antineuronal autoantibodies yet never develop a tumor, with the main examples including LEMS, neuromyotonia, and LE. Therefore, the presence of antibodies does not prove the presence of a tumor. Some autoantibodies are present in low titers in cancer patients without accompanying neurological manifestations. Patients with suspected paraneoplastic syndromes may be asymptomatic or have “atypical” or poorly characterized antibodies that are not detected through routine diagnostic tests. Some initially “antibody-negative” patients later test positive for ANeAs. Therefore, a negative antibody result does not exclude the possibility of a paraneoplastic disorder and the presence of an underlying neoplasia [2].

Although paraneoplastic antibodies are synthesized intrathecally, pathogenicity has been demonstrated only for those directed against antigens located on the cell surface. A distinct mechanism is observed in PCD associated with Hodgkin lymphoma, since the target antigens of the respective anti-Tr and anti-mGluR1 autoantibodies are not expressed in the tumor tissue of Hodgkin lymphoma. In this case, the main mechanism is immune dysregulation and a potential etiological role of viral infections [34].

Aydin et al. [35] analyzed the prognostic factors associated with ANeA seropositivity using detection methods such as immunofluorescence, IB, and immunocytochemistry. Patients exhibited antibodies to different target neuronal antigens, including NMDAR and Zic4. Detection of antibodies to extracellular or synaptic target antigens, but not the presence of a tumor, cranial MRI lesions, or oligoclonal bands, was associated with a good prognosis and response to treatment. NMDAR was one of the most prevalent ANeAs in this screening study; therefore, this antibody type has been identified as the most important prognostic factor in clinical cases that are positive for ANeAs [35].

The diagnostic value of the presence of ANeAs in serum was investigated in patients with suspected PND (group 1) and compared with three control groups. In group 1, nuclear ANeAs were detected in eight patients (38%). Six patients had SCLC, one had ovarian cancer, and in one patient, no tumor was identified. Neurological symptoms preceded the diagnosis of cancer in five out of these eight patients. ANeAs were found in the serum of a very small number of patients (2%) in one of the control groups. These data show a moderate sensitivity of 38% but a high specificity of 98.6% for the presence of nuclear ANeAs in suspected PND [36].

The main aim of this scientific work is to analyze two patients with a clinical course, neuroimaging findings, and ANeAs suggestive of PCD and to perform long-term follow-up to facilitate early diagnosis, which is associated with better prognosis and disease outcome. Moreover, if the follow-up neuroimaging diagnostics shows a tumor-negative result, these case series could possibly represent non-paraneoplastic cerebellar degeneration.

## 2. Case Presentation

### 2.1. Patient Information

Patient 1 (38-year-old female) was admitted to the Neurology Clinic of a University Hospital in Plovdiv in August 2023 with complaints of twitching of the right hand and leg and in the neck. After several days, this patient was transferred to the ICU clinic and thereafter transferred back to the Neurology Clinic.

Patient 2 (66-year-old male) was admitted to the Neurology Clinic of a University Hospital in Plovdiv in November 2023 with complaints of gait disturbances, weakness in the hands, and severe disability. The patient had previously been taking corticosteroids, Imuran, Cavinton, and Milgamma.

### 2.2. Clinical Findings

Patient 1 presented with a severe clinical course characterized by rhythmic twitching of the right limbs and neck with the characteristics of focal motor clonic seizure with a preserved level of consciousness.

Patient 2 presented with progressive gait disturbances, weakness in the hands, and progressive functional decline, culminating in complete disability over the preceding 18–20 months. Presence of intrathecal inflammatory activity. Additional symptoms included diplopia, deterioration in general functionality and bulbar function, and impaired vision. The overall clinical picture was consistent with chronic inflammatory autoimmune pathology with polyneuropathy and possible CNS involvement.

### 2.3. Timeline

Because of clinical signs suggestive of PNSs, both patients were tested for ANeAs. Serum and CSF were collected from the patients and examined at the Research Institute of Medical University-Plovdiv, Bulgaria.

Both patients were tested twice for ANeAs over a six-month period (between August 2023 and January 2024).

A commercial indirect immunofluorescence test kit (Autoimmune Encephalitis Mosaic 6, cat. No. FA 112d-1005-1, EUROIMMUN, Lübeck, Germany) was used for the detection of ANeAs targeted against cell-surface neuronal antigens (NMDAR, CASPR2, AMPAR1/2, LGI1, DPPX, GABAbR), following the manufacturer’s instructions.

A commercial immunoblot test kit (EUROLINE Paraneoplastic Neurological Syndromes 12 Ag, cat No. DL 1111-1601-7G, EUROIMMUN, Lübeck, Germany) was used for the detection of ANeAs targeted against intracellular neuronal antigens (Hu, Ri, Yo, Ma2/Ta, amphiphysin, recoverin, Zic4, titin, GAD65, CV2, SOX1, Tr), following the manufacturer’s instructions. Anti-Zic4 ANeAs were measured by means of IB.

Patient 1 had serum anti-Zic4 antibodies of high intensity = 54 and was positive for anti-NMDAR antibodies in serum and CSF in October 2023. The same patient was tested again 2 months later (August 2023) for ANeAs and then again for anti-Zic4, with high antibody intensity = 105 (Table 1).

Patient 2 had serum anti-Zic4 antibodies of low intensity = 19 in January 2024. The same patient was first tested for ANeAs 2 months prior (November 2023) and, at that time, exhibited anti-Zic4 positivity of moderate intensity = 48 (Table 1).

The first autoantibody (anti-Zic4) was of high band intensity and the second one (anti-NMDAR) exhibited a high level of immunofluorescence. Anti-Zic4 antibodies are associated with PCD; anti-NMDAR antibodies are associated with autoimmune anti-NMDAR encephalitis.

The intensity of anti-Zic4 in the first patient was low, whereas in the second patient, it was high. These autoantibodies are associated with cerebellar degeneration.

**Figure 1:**
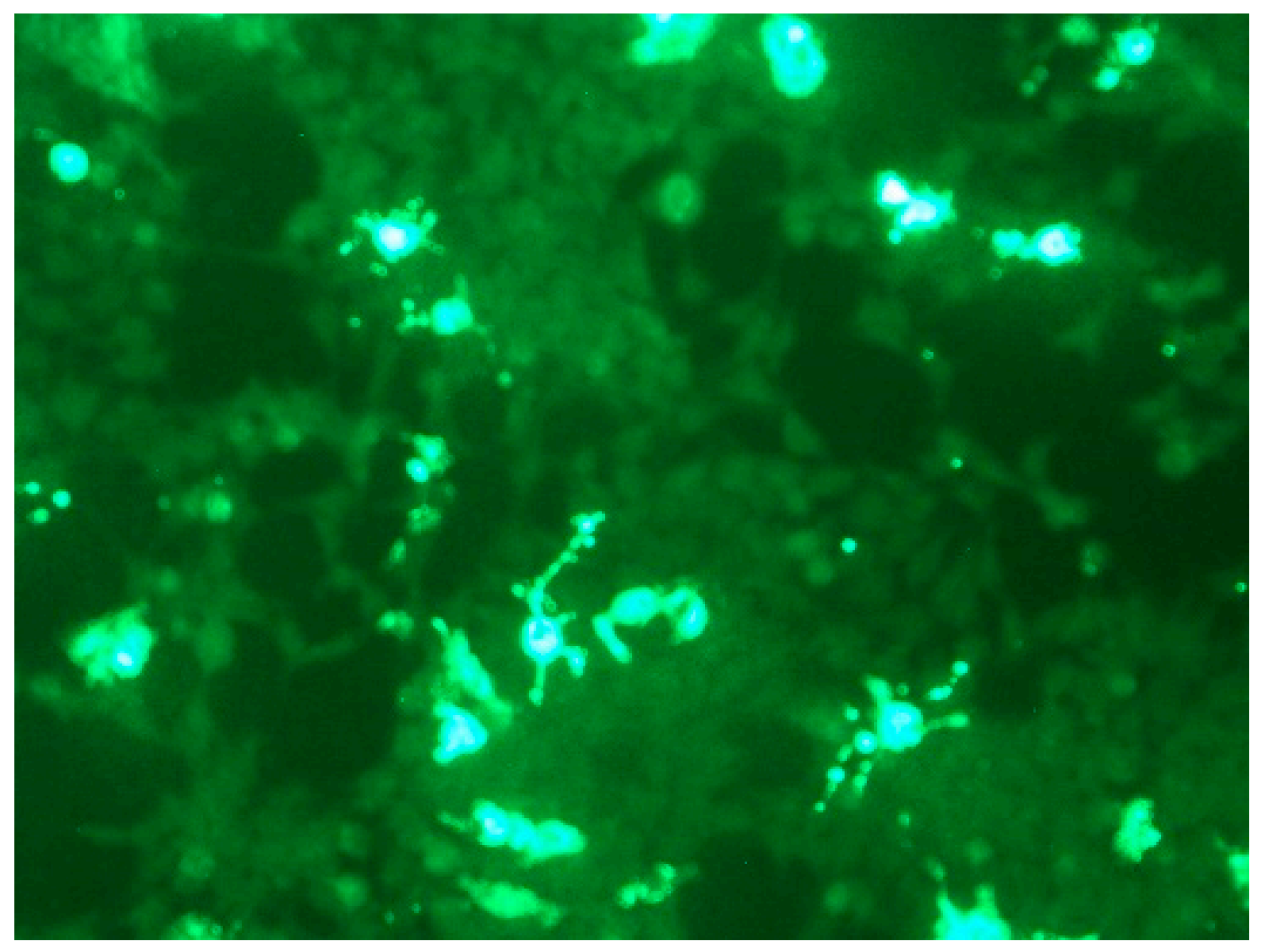
Patient 1, anti-NMDAR positive, October 2023.

**Figure 2:**
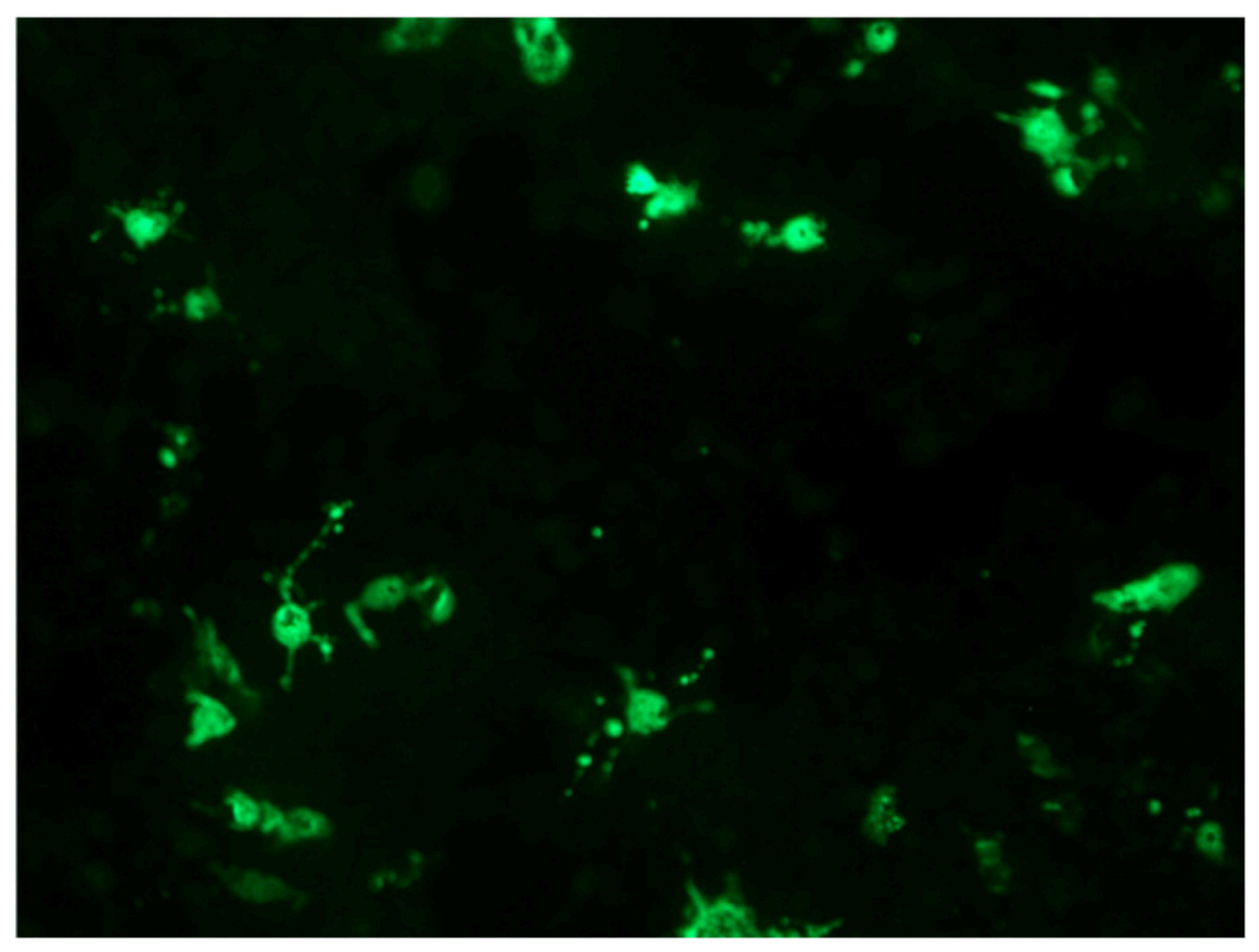
Patient 1, anti-NMDAR positive, October 2023.

**Figure 3:**
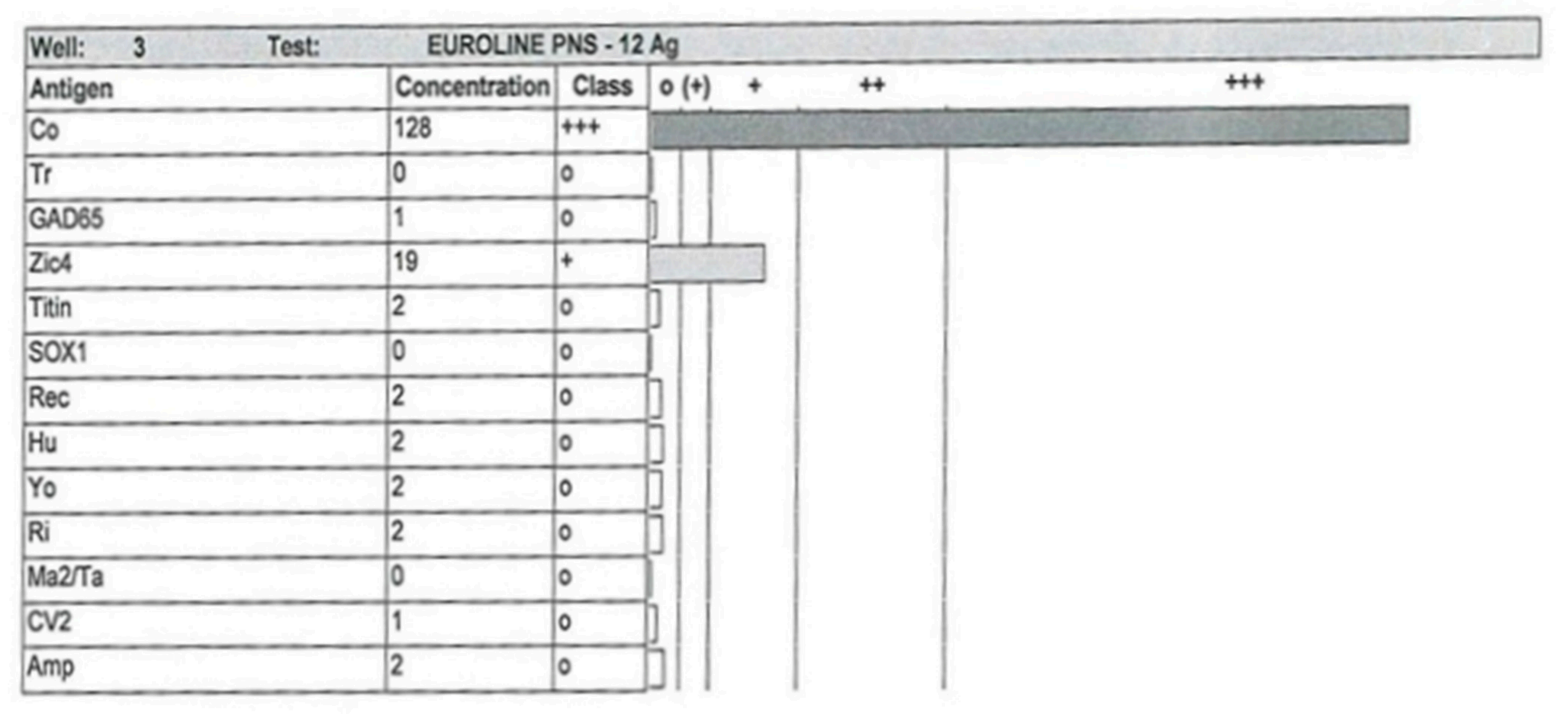
IB image of Patient 2, January 2024.

**Figure 4:**
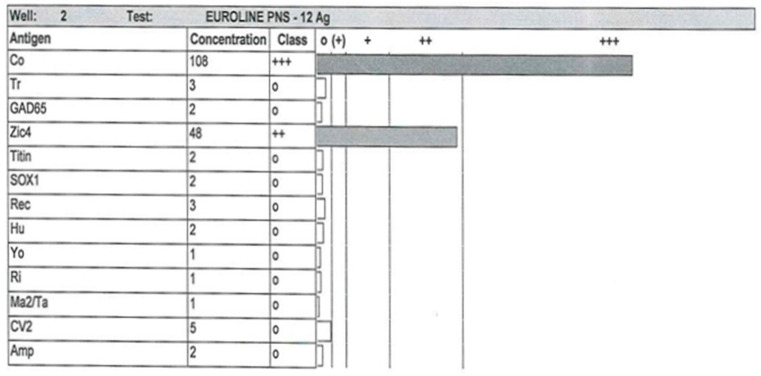
IB image of Patient 2, November 2023.

**Figure 5:**
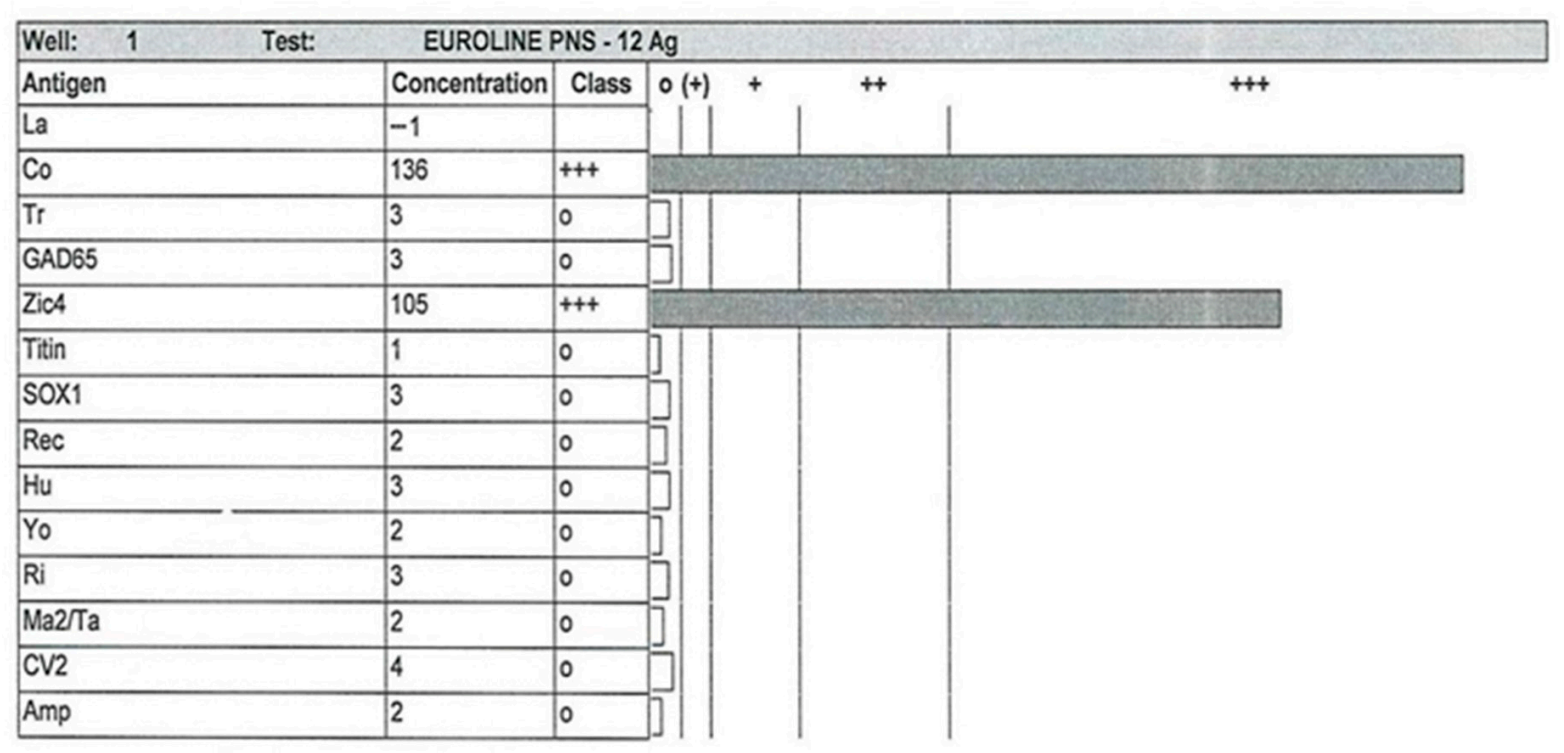
IB image of Patient 1, August 2023.

**Figure 6:**
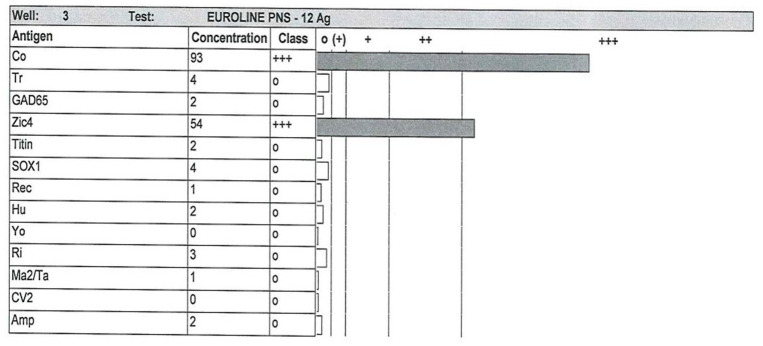
IB image of Patient 1, November 2023.

The IIFA and IB images of Patient 1 and Patient 2 are shown in Figure 1, Figure 2, Figure 3, Figure 4, Figure 5 and Figure 6.

Reference ranges for anti-Zic4 ANeA band intensity:

➢ intensity 0–5—negative result/-/;
➢ intensity 6–10—borderline result—interpreted as a negative result/-/;
➢ intensity 11–25—weak positive result/+/;
➢ intensity 26–50—moderate positive result/++/;
➢ intensity > 50—high positive result/+++/.

### 2.4. Diagnostic Assessment

#### 2.4.1. Neurological Status

Patient 1—1. Extrapyramidal syndrome—orofacial dystonias; 2. pyramidal syndrome—pathologically lively tendon reflexes in the upper limbs, Babinski positive, Chadok positive (left), and severe quadriparesis; 3. polyneuritic syndrome—severely weakened to absent tendon reflexes in the lower limbs symmetrically.

Patient 2—severely disabled patient. 1. Polyneuritic syndrome—disability; quadriparesis—moderate in the proximal area of the legs and severe in the feet and mild to moderate in the hands; tendon-supraosseous reflexes—absent; hypesthesia in the legs and hands with disturbed deep sensation in the feet; 2. brainstem syndrome with minimal internuclear ophthalmoparesis; discrete dysphagia.

#### 2.4.2. Laboratory Testing

The laboratory deviations in ANeA, CBC, DBC, coagulogram, biochemical examinations, blood gas analysis, and CSF diagnostics are shown in Table 1, Table 2, Table 3 and Table 4.

Furthermore, Patient 1 had elevated tumor markers: CA 19–9–221 U/mL (reference range 2.5–33 U/mL) and CA125–105.1 IU/mL (0–35 IU/mL). This finding prompted clinical suspicion of an oncological disease. We recommend screening for serum CA 19–9 and CA125 in patients with such a clinical course of PCD, because the most common malignancies associated with PCD are SCLC, in addition to ovarian tumors and, in some cases, gastrointestinal tumors.

Because of the clinical signs and symptoms, suggestive of PNSs, neuroimaging diagnostic studies were performed. During follow-up examinations, no tumor was found on neuroimaging studies and CT and MRI scans.

Both patients also presented serious abnormalities in their laboratory parameters—complete blood count (CBC), differential blood count (DBC), and coagulogram (Table 2), CSF immunological examinations and blood gas analysis (Table 3), and biochemistry tests (Table 4).

No routine cerebrospinal fluid (CSF) findings were available for either Patient 1 or Patient 2.

The abnormalities in their CSF diagnostics were presented as a significant increase in the total level of IgG and a significant decrease in leucocyte level in CSF. Furthermore, these individuals exhibited increased levels of total protein in CSF and both patients exhibited decreased levels of pCO_2_ from their blood gas analysis.

The CBC of Patient 1 indicated a significant decrease in red blood cells, hemoglobin, and hematocrit (anemic syndrome) and a significant decrease in platelets and plateletcrit (thrombocytopenia) and the coagulogram showed elevated fibrinogen. The biochemical abnormalities in Patient 1 included a significant decrease in urea, albumin, and total protein levels, a non-significant decrease in creatinine and K^+^ and Ca^2+^ levels, a significant increase in procalcitonin (PCT) levels, and a non-significant increase in ASAT and ALAT levels. The CRP level of Patient 1 was significantly elevated. Blood gas analysis of Patient 1 indicated increased pH and decreased pCO_2_. CSF diagnostics of Patient 1 indicated a significant increase in IgG and erythrocytes, a non-significant increase in total protein levels, and a significant decrease in leucocyte level in CSF.

The CBC of Patient 2 indicated the presence of leucocytosis; the DBC of Patient 2 indicated a significant decrease in monocyte and lymphocyte percentages, a non-significant decrease in the absolute count of lymphocytes, and a significant increase in the absolute count and percentage of neutrophils. The biochemical abnormalities in Patient 2 include a non-significant increase in glucose level. Blood gas analysis of Patient 2 indicated increased pCO_2_ and decreased pO_2_ levels. CSF diagnostics of Patient 2 indicated a significant increase in IgG level and total protein level, an insignificant increase in glucose level, and a significant decrease in leucocyte level in CSF.

In a comparison of the laboratory data, the CSF immunological examinations (IgG, Glu, Leuc, U-PROT, and Erys) of Patient 2 revealed more deviations than in Patient 1; the biochemical examinations of Patient 1 revealed more deviations than in Patient 2. The CBC and coagulogram results indicate more deviations in Patient 1 compared with Patient 2. The DBC is abnormal only in Patient 2. The blood gas analysis parameters are abnormal in both Patient 1 and Patient 2.

In a combined analysis of the laboratory results, IgG-CSF and T-PROT-CSF were found to be elevated in both Patient 1 and Patient 2. Leuc-CSF decreased in both Patient 1 and Patient 2. Glu-CSF in Patient 2 increased, whereas Glu-CSF in Patient 1 was within normal limits (WNL). Ery-CSF in Patient 1 was elevated, while Ery-CSF in Patient 2 was WNL.

#### 2.4.3. Image Diagnostics

Chest CT scan of Patient 1 showed bilateral solid nodules suggestive of metastases and their MRI findings showed involvement of the cerebellum and the frontal lobe of the cerebrum. The MRI findings of Patient 2 revealed lesions suggestive of cerebellar degeneration.

The bilateral solid nodules in Patient 1 are localized intraparenchymally and subpleurally.

#### 2.4.4. Electromyography (EMG)

EMG examination of Patient 2 confirmed severe polyneuropathy.

#### 2.4.5. Electroencephalography (EEG)

EEG examination of Patient 1 showed absence of focal and paroxysmal abnormalities; the EEG of Patient 2 was WNL, with no abnormalities detected.

#### 2.4.6. General Pathology

Biopsy material of Patient 1—no tumor cells were detected.

Patient 1 did not have a detected malignant neoplasia (CT, MRI and PET-CT were tumor-negative).

#### 2.4.7. Diagnosis

Patient 1—Grand mal seizures and generalized epilepsy with structural etiology, occurring with generalized bilateral myoclonic seizures.

Patient 2—Inflammatory polyneuropathy. Diseases of the cranial nerves, nerve roots, and plexuses and vertebrogenic pain syndromes in patients over 18 years of age. Comorbidities—Surgical intervention for herniated disc at the level of L5-C1. Spinal canal stenosis.

#### 2.4.8. Therapeutic Intervention

Patient 1—Sodium valproate, Levetirazetam, Sulbactam/Cefoperazone, Amikacin, ICU–Methylprednisolone.

Patient 2—Ringer solution, Dexamethazone, Omeprazole, Torasemide, 0.9% Sodium chloride, Methylprednisolone.

Plasma exchange was not considered.

## 3. Discussion

Patient 1, who was 38 years old, tested positive for serum anti-Zic4, which is associated with cerebellar degeneration (evidenced by neuroimaging diagnostics—MRI); furthermore, high band intensity of anti-Zic4 correlated with severely impaired neurological status of the patient at the time of examination—focal motor clonic seizure (orofacial dystonias) with a preserved level of consciousness; pyramidal syndrome; severe quadriparesis; and polyneuritic syndrome. In the same patient, anti-NMDAR, detected in serum and CSF, is associated with autoimmune anti-NMDAR encephalitis, which was evidenced by the diagnostic criteria, suggested by Graus et al. [37] (altered neurological status—autonomic dysfunction, severely impaired general condition, dyskinesias, and epileptic seizures); moreover, high intensity of anti-NMDAR was observed via IIFA. In the same patient, serious deviations in CBC, DBC, biochemical tests, blood gas analysis, and CSF diagnostics were also observed. There are no literature sources describing such a clinical case with the presence of both anti-Zic4 and anti-NMDAR ANeA, combined with MRI findings suggestive of cerebellar degeneration and serious deviations in laboratory parameters and CSF diagnostics.

In the study by Qiao et al. [38], the coexistence of multiple ANeAs in patients with AE may cause a superimposition and diversification of clinical manifestations, potentially suggestive of an underlying malignancy [38].

The focal motor clonic seizure observed in Patient 1 correlated with the positivity of anti-Zic4 ANeA, which is supported by a study of Iranian patients with autoimmune encephalitis in which anti-Zic4 antibodies were identified in 10.3% of the cohort, with 75% of those patients experiencing seizures [39]. Furthermore, focal seizures, including motor symptoms, can occur within the context of Zic4-associated paraneoplastic encephalitis [20].

The brainstem dysfunction observed in Patient 2, a 66-year-old, with progressive cerebellar ataxia, severe imbalance, gait ataxia, and dizziness, correlates with the positivity of anti-Zic4 ANeA because the hallmark of anti-Zic4 antibody-associated syndrome is subacute cerebellar dysfunction, which may occur in isolation or with brainstem involvement (rhombencephalitis) [18].

Anti-Zic4 seropositivity is associated with PCD, with the most frequently associated tumor being SCLC; in this case, screening for the corresponding tumor is required [40]. Another tumor type associated with anti-Zic4 and PCD is primary pulmonary lymphoma, described in a 67-year-old man with bilateral eyelid ptosis, dysarthria, dysphagia, and hypophonia. MRI data revealed a hyperintense lesion, indicating cerebellar atrophy. Clinically, an ataxic syndrome with involvement of the first and second motor neurons, autonomic dysfunction, and diffuse encephalopathy was recorded [12]. Anti-Zic4 antineuronal antibodies are associated with PCD, most often in patients with SCLC [18].

Both ANeA panels (Paraneoplastic Neurological Syndromes 12 Ag and Autoimmune Encephalitis Mosaic 6) are included in the examination because, in some cases, non-paraneoplastic autoimmune encephalopathies can present with the symptoms observed in the patients included in this study. The advantage of using these ANeA kits is the simultaneous assessment of multiple ANeAs.

No tumor was detected on CT, MRI, and PET-CT; therefore, Patient 1 and Patient 2 should undergo long-term follow-up because, in some cases, PNSs can precede the diagnosis of cancer by 4 years. The clinical signs and symptoms observed in these two patients (orofacial dystonias, pyramidal syndrome, polyneuritic syndrome, and brainstem syndrome) are considered to be paraneoplastic. Therefore, follow-up examinations are required to screen for eventual malignant neoplasia.

In patients without cancer but with a PNS, tumor detection is an important but sometimes diagnostically difficult process. As there is an effective immune response against the tumor, it may initially remain small and localized. If specific autoantibodies are present, they direct the physician to a specific tumor localization. PNSs associated with malignant tumors typically manifest months or even years before the oncological disease is detected using traditional diagnostic techniques. This fact allows us to consider paraneoplastic ANeAs as promising biomarkers for early cancer diagnosis [41]. Follow-up of the patients is required because, in some cases, the initial symptoms of the paraneoplastic neurological manifestations precede the detection of the respective tumor by four years [42,43,44].

The gold standard for anti-NMDAR autoimmune encephalitis is testing with anti-NMDAR antibodies. The test kit Autoimmune Encephalitis Mosaic 6 possesses very high diagnostic sensitivity and specificity; therefore, it is used to diagnose anti-NMDAR autoimmune encephalitis.

The novelty of this study lies in the presentation of the first case of a possible non-paraneoplastic anti-Zic4 and anti-NMDAR positive patient (Patient 1) with clinical signs suggestive of cerebellar degeneration and autoimmune encephalitis and neuroimaging diagnostics, confirming pathological involvement of the cerebellum. Moreover, the chest CT scan of Patient 1 showed bilateral solid nodules suggestive of metastases. The MRI findings of Patient 2 also showed involvement of the cerebellum. These findings necessitate extensive follow-up of both individuals in order to detect a possible malignant neoplasia and to start a therapeutic course, because, in some cases, PNSs can precede the detection of an oncological disease by years.

## 4. Conclusions

Panels of ANeAs (directed against intracellular antigens and cell surface antigens), which enable simultaneous multiple onconeuronal antigen testing, have been used in targeted patients with cerebellar syndrome suggestive of PNSs. In the first patient (Patient 1) in this study, anti-Zic4 was associated with cerebellar degeneration; furthermore, high band intensity of anti-Zic4 correlated with the patient’s severely impaired neurological status at the time of examination. In the same patient, anti-NMDAR was associated with autoimmune encephalitis, which was evidenced by their altered neurological status. Early diagnosis of anti-NMDAR encephalitis leads to initiation of immunomodulatory therapy and improves clinical outcomes. The above patient may represent the first case of possible non-paraneoplastic cerebellar degeneration in a patient positive for both anti-Zic4 and anti-NMDAR. Moreover, bilateral solid nodules suggestive of metastases were evidenced by the CT scan of Patient 1. In the other patient (Patient 2), MRI also showed lesions, suggestive of cerebellar degeneration. However, extensive follow-up of both patients is required because, in some cases, PNSs can precede the detection of the respective oncological disease by years.

## Figures and Tables

**Table 1:** Anti-Zic4 ANeAs of Patient 1 and Patient 2.

ANeA	Patient 1 August 2023	Patient 1 October 2023	Patient 2 November 2023	Patient 2 January 2024
Serum anti-Zic4 (band intensity)	105	54	48	19

**Table 2:** CBC, DBC, and coagulogram results.

Parameters	Patient 1	Patient 2	Reference Ranges
Fbg	4.77 g/L	-	2.0–4.5 g/L
HGB	70 g/L	-	120–160 g/L
RBC	2.53 × 10^12^/L	-	3.9–5.3 × 10^12^/L
WBC	-	12.17 × 10^9^/L	3.5–10.5 × 10^9^/L
HCT	0.22 L/L	-	0.36–0.47 L/L
RDW	18.5%	-	11–16%
PLT	57 × 10^9^/L	-	140–400 × 10^9^/L
Plateletcrit	0.0063%	-	0.109–0.485%
APTT	-	22.8 s	23–35 s
CRP	177 mg/L	-	0–10 mg/L
Abs. count of Ly	-	1.12 × 10^9^/L	1.3–3.9 × 10^9^/L
% Ly	-	7.2%	22–48%
% Mo	-	1.7%	5.8–11.8%
Abs. count of Neu	-	14.24 × 10^9^/L	2.2–4.5 × 10^9^/L
% Neu	-	90.7%	22–45%

**Table 3:** CSF diagnostic parameters and blood gas analysis.

Parameters	Patient 1	Patient 2	Reference Ranges
IgG-CSF	70.37 mg/L	114.22 mg/L	0–40 mg/L
Leuc-CSF	11 × 10^6^/L	15 × 10^6^/L	20–25 × 10^6^/L
U-CSF-PROT	0.61 g/L	0.9 g/L	0.15–0.45 g/L
Glu-CSF	-	4.1 mmol/L	2.2–3.9 mmol/L
Erys-CSF	2 × 10^9^/L	-	0–0.15 × 10^9^/L
pH	7.52	-	7.35–7.45
pO_2_	-	68 mmHg	83–108 mmHg
pCO_2_	28 mmHg	48 mm/Hg	35.5–44.7 mmHg

**Table 4:** Biochemical tests.

Parameters	Patient 1	Patient 2	Reference Ranges
K^+^	2.6 mmol/L	-	3.5–5.6 mmol/L
Ca^2+^	1.93 mmol/L	-	2.12–2.62 mmol/L
ASAT	39 U/L	-	0–36 U/L
ALAT	38 U/L	-	0–35 U/L
Glu	-	8.9 mmol/L	2.8–6.1 mmol/L
ALB	19 g/L	-	35–52 g/L
T-PROT	39.1 g/L	-	60–83 g/L
PCT	5.69 ng/mL	-	0.02–0.30 ng/mL
CREA	43 μmol/L	-	44–96 μmol/L
UREA	1.3 mmol/L	-	2.6–7.2 mmol/L

## Data Availability

The original contributions presented in this study are included in the article. Further inquiries can be directed to the corresponding author.
